# In vitro assessment of experimental bioactive tooth gel formulas containing L-arginine, ginger and eggshell for remineralization and anti-bacterial effect

**DOI:** 10.1038/s41598-026-54994-z

**Published:** 2026-06-04

**Authors:** Reham S. Saleh, Hadeer A. El-Hashemy, Hanaa M. Elgamily

**Affiliations:** 1 Restorative and Dental Materials Department, Oral and Dental Research Institute, National Research Centre, Cairo, Egypt; 2 Department of Pharmaceutical Technology, National Research Centre, Cairo, Egypt

**Keywords:** White spot lesions, Remineralization, Antibacterial, L-arginine, Ginger and eggshell, Health care, Medical research, Microbiology

## Abstract

The present study evaluates the effect of different novel tooth gel formulations combining L-arginine, ginger and eggshell on artificially demineralized enamel. This study used 50 bovine enamel specimens, divided into five groups according to the used toothpaste (*n* = 10): G1: Elmex Pro Argin (commercial positive control containing L-arginine), and four experimental groups; G2: ginger extract, G3: experimental eggshell + ginger, G4: experimental L-arginine + ginger, and G5: experimental L-arginine + eggshell + ginger. White-spot lesions were induced then the tested formulations were applied twice daily for one month. Evaluations at baseline, post-demineralization, and post-remineralization included Vickers microhardness (VMH), Scanning electron microscope (SEM), Energy Dispersive X-ray Analysis (EDX), elemental mapping and Confocal Laser Scanning Microscopy (CLSM). Also, antibacterial activity was assessed against commensal and cariogenic bacteria. After remineralization, all experimental groups showed a statistically significant increase in microhardness compared to their corresponding demineralized values (*P* < 0.001). Arginine-containing formulations (G4 and G5) showed statistically significant higher microhardness values and Calcium content following EDX analysis compared to the non-arginine formulations (G2 and G3) (*P* < 0.05). In agar well diffusion assay, the arginine-containing formulations (G1, G4, and G5) produced statistically significantly larger inhibition zones against both cariogenic and commensal bacterial species compared with the arginine free formulations (G2 and G3) at *P* < 0.05. The synergy of L-arginine with ginger and eggshell extract provides enhanced enamel remineralization, and antimicrobial efficacy, positioning these natural tooth gel formulations as promising alternatives or adjuncts to conventional fluoride-based products.

## Introduction

Dental caries remains a prevalent global health concern, resulting from the demineralization of tooth enamel due to acidic by-products from bacterial metabolism within dental biofilms. This demineralization can be counteracted by remineralization processes, which restore lost minerals to the enamel, thereby preventing the progression of carious lesions^1^.

L-arginine, a naturally occurring amino acid, has garnered attention for its role in oral health. It serves as a substrate for arginolytic bacteria, leading to the production of ammonia, which neutralizes plaque acids and elevates oral pH. This alkalinization fosters an environment conducive to remineralization and inhibits the growth of acidogenic bacteria like *Streptococcus mutans*. Recent studies have demonstrated that arginine, especially when combined with fluoride, enhances enamel remineralization and increases fluoride uptake when combined with other remineralizing agents^2–4^.

Fluoridated toothpastes are widely consumed in the market due to the well-known remineralizing effect of fluoride. In contrast, it has been reported that some problems can be raised due to the chronic low‑level fluoride exposure. Also, it has been documented that there is a noticeable increase in the prevalence of dental fluorosis even in non-fluoridated areas. Moreover, the ability of fluoride to remineralize the deepest part of the demineralized enamel lesion is limited. This brings attention to the need for an effective remineralizing alternative with a wider safety margin^4–6^.

Fluoride remains the gold standard in caries prevention due to its well-documented ability to enhance remineralization and inhibit demineralization. However, growing interest in fluoride-free or adjunctive bioactive formulations has encouraged the exploration of alternative strategies that may complement or, in specific cases, substitute conventional approaches. Among these, L-arginine has been shown to modulate plaque pH and promote a less cariogenic biofilm. Although synergistic effects between arginine and fluoride have been reported, the present study aims to evaluate the independent efficacy of arginine-based fluoride-free formulations to better understand their individual contribution to enamel remineralization and antibacterial activity^2–5^.

In parallel, natural substances such as ginger and eggshell-derived hydroxyapatite have been explored for their potential benefits in oral care. Ginger exhibits notable antibacterial properties against oral pathogens, contributing to reduced biofilm formation and acid production^7,8^. Eggshell powder, rich in calcium carbonate, has been shown to aid in enamel remineralization and improve surface microhardness, offering a cost-effective alternative to conventional agents^9,10^.

However, up till now there isn’t any study assessed the synergistic effect of arginine with either ginger or eggshell on enamel remineralization and their antibacterial effect. Given the individual benefits of L-arginine, ginger, and eggshell-derived compounds, this study aims to evaluate their combined effects in tooth gel formulations. Specifically, it investigates their efficacy in remineralizing artificially demineralized enamel as well as their antibacterial activity against cariogenic bacteria. By exploring these combinations, our study seeks to identify synergistic effects that could enhance oral health outcomes.

Thus, the null hypothesis assumes that none of these experimental naturally based gel formulations could enhance remineralization of the demineralized enamel nor inhibit the anti-cariogenic bacteria in comparison to the commercially used L- arginine toothpaste.

### Materials and methods

#### Ethical approval

This in vitro study was performed in line with the ethical guidlines of the Medical Research Ethics Committee (MREC) of the National Research Centre under approval 19–249 on March 2022.

## Study design and grouping

This study was designed as a randomized, double-blind in vitro experimental investigation. The sample size (*n* = 10 specimens per group) was determined based on previously published in vitro enamel remineralization and antibacterial studies, which commonly employed 8–10 specimens per group to achieve reliable detection of intergroup differences using one-way ANOVA analysis^11–13^. As this was an exploratory in vitro study, no formal a priori power calculation was performed.

Randomization was performed using a computer-generated random sequence, and both the examiner and statistician were blinded in all outcome assessments to eliminate bias.

A total of fifty sound bovine teeth were selected and randomly allocated into five experimental groups (*n* = 10) according to the applied toothpaste formulation, as follows:

- **Group 1 (G1)**: L-arginine–based commercial toothpaste. (Elmex Pro Argin.

positive control).

- **Group 2 (G2)**: Ginger extract–based experimental tooth gel.
- **Group 3(G3)**: Combined ginger and eggshell extract–based experimental.

Tooth gel.

- **Group 4(G4)**: Ginger extract–based experimental tooth gel supplemented with.

8% w/v L-arginine.

- **Group 5(G5)**: Combined ginger and eggshell extract–based experimental.

Tooth gel supplemented with 8% w/v L-arginine.

## Preparation of the natural experimental toothpastes

### Preparation of natural extracts

Ginger extract and the combined eggshell–ginger extract were prepared according to the protocol described in a previous study by Saleh et al.^13^. Both Ginger and egg were obtained from a local market. Ginger was cleaned with distilled water in order to remove any dirt then it was peeled into thin slices. While the eggshell powder was acquired through the calcination process in accordance with the World Intellectual Property Organization’s protocol (WO/2004/105912: Method of generating eggshell powder)^13^.

These extracts were selected based on their proven remineralization potential on demineralized enamel and their antibacterial activity against cariogenic bacteria.

### Preparation of tooth gel formulations

Four experimental gel formulations were prepared and subsequently compared with the commercially available L-arginine toothpaste (Elmex Sensitive Professional PRO-ARGIN, GABA International AG, Switzerland) which was used as a positive control. It contains 8% arginine as an active ingredient, while other ingredients are Calcium Carbonate, Aqua, Sorbitol, Bicarbonate, Sodium Lauryl Sulfate, Aroma, Sodium Monofluorophosphate, Cellulose Gum, Sodium Bicarbonate, Tetrasodium Pyrophosphate, Sodium Saccharin, Benzyl Alcohol, Xanthan Gum, Limonene, CI 77,891.

The base vehicle used in all formulations consisted of Carbopol 940 dispersed in water, then it was gradually dispersed into the mixture under gentle agitation to allow complete polymer hydration. Afterward, the gel was neutralized using triethanolamine (TEA) which was added dropwise until a clear, uniform gel consistency was obtained. Afterwards, each formulation was prepared by incorporating the designated active ingredients into the base vehicle at a 1:1 volume ratio under continuous mechanical stirring until a homogeneous mixture was achieved.

All prepared formulations were stored under refrigerated conditions until use to preserve chemical stability.

### pH measurement

The pH of each toothpaste formulation was measured directly on the prepared gels using a calibrated digital pH meter prior to application. Measurements were performed in triplicate, and mean values were recorded to ensure consistency of the prepared formulations.

The pH values of all tested formulations ranged from 6.5 to 7.2 (Table 1), which falls within the physiologically acceptable range for oral applications.

Formulations containing L-arginine (G1, G4, and G5) confirming the alkalizing effect of L-arginine. Among extract-based formulations, the ginger extract–based tooth gel (G2) can be attributed to the mild acidity of ginger and the buffering capacity of calcium carbonate derived from eggshell.

Importantly, none of the formulations exceeded the neutral tolerance range, and all pH variations remained within the ± 5% threshold considered clinically insignificant for oral safety. These findings indicated satisfactory chemical stability and biocompatibility of the developed tooth gel formulations, particularly those supplemented with L-arginine.

**Table 1:** pH Values of the Commercial Tooth paste and the Experimental tooth gel Formulations (mean ± SD).

Group	Formulation	pH
G1	L-arginine based commercial toothpaste (Positive control)	7.1 ± 0.4
G2	Ginger extract–based tooth gel.	6.5 ± 0.5
G3	Combined Ginger + eggshell extract tooth gel.	6.7 ± 1.3
G4	Ginger extract + 8% w/v L-arginine tooth gel.	6.9 ± 0.5
G5	Combined Ginger + eggshell extract + 8% w/v L-arginine tooth gel.	7.2 ± 1.2

## Teeth selection and specimen preparation

For this study, a total of fifty sound bovine anterior teeth were collected from a local butcher. Soft tissue remnants and debris were removed using a hand scaler, followed by thorough rinsing under running water. All teeth were carefully examined under magnification to exclude specimens presenting cracks, hypoplasia, surface defects, or white spot lesions.

Disinfection was carried out by immersing the teeth in 5% sodium hypochlorite solution for 5 days, after which they were stored in deionized water until the start of the experiment^14^. Afterwards, each tooth was subjected to prophylaxis polishing using rotary rubber cups with pumice slurry for a minute and washed with distilled water^15^. Then teeth were decoronated, and longitudinal sectioning was performed using a diamond saw under continuous water irrigation to obtain defect-free labial enamel slices with 3 × 3 mm window^15,16^.

Each enamel specimen surface was standardized by sequential polishing using silicon carbide abrasive papers of decreasing grit sizes (e.g., 600, 800, and 1200 grit) under water cooling to obtain a flat, smooth, and standardized surface suitable for microhardness testing, following horizontally positioned at the center of sectional teflon mould (4 cm diameter) and embedded in auto-polymerized acrylic resin. The acrylic resin was allowed to set for one hour to ensure complete polymerization before further experimental procedures^13,14^.

## Formation of artificial enamel demineralization

Artificial white spot enamel lesions were induced using a pH-cycling model over five consecutive days. Each cycle consisted of 3 h of demineralization followed by 21 h of remineralization in artificial saliva. Specimens were rinsed with distilled water after each complete cycle, and both the demineralizing solution and artificial saliva were refreshed every 24 hours^13, 17–19^.

### Solutions composition

**Artificial saliva** was prepared with the following composition:

- 1.5 mmol/L Ca(NO₃)₂·4 H₂O.
- 0.9 mmol/L NaH₂PO₄·2 H₂O.
- 150 mmol/L KCl.
- 0.1 mol/L Tris buffer.
- 0.03 ppm fluoride.

The pH of artificial saliva was adjusted and maintained at 6.57.

**Demineralizing solution** consisted of:

- 2.2 mM calcium chloride (CaCl₂·2 H₂O).
- 2.2 mM monosodium phosphate (NaH₂PO₄·7 H₂O).
- 0.05 mM lactic acid.

The solution pH was adjusted to 4.5 using 50% sodium hydroxide (NaOH).

### Application of different formulations

Application of the experimental tooth gels and the commercial control paste was performed using manual brushing twice daily for one month. Separate medium-bristle toothbrushes were allocated to each group to prevent cross-contamination. All brushing procedures were carried out by a single calibrated operator applying standardized light manual pressure (approximately 200 g) to minimize variability in brushing force. In addition, toothbrushes were replaced weekly to maintain consistent bristle stiffness. Each brushing session lasted 2–3 min using circular motions to ensure uniform material application. Following brushing, specimens were rinsed with deionized water and stored in artificial saliva until the subsequent brushing cycle^20,21^.

Enamel specimens were evaluated at baseline, after demineralization, and after remineralization for surface microhardness, Scanning electron microscope (SEM), Energy Dispersive X-ray Analysis (EDX), elemental mapping and Confocal Laser Scanning Microscopy (CLSM).

## Remineralization assessment

### Surface microhardness evaluation (VMH)

All specimens were assessed for Digital Enamel surface microhardness using a Digital Vickers Microhardness Tester (NEXUS 4000™, INNOVTEST, Model 4503, The Netherlands) at 20× magnification. A load of 200 g was applied for 15 s at each indentation site. Three indentations were made per specimen at each evaluation stage, and the mean Vickers hardness number (VHN) was calculated^15^.

### Scanning electron microscope (SEM)

The enamel surfaces were examined for any structural changes occurring after the experimental stages and recorded by SEM (Quanta FEG 250, FEI, Hillsboro, OR, USA) at ×10,000 magnification. The specimens were not subjected to any preparation before scsnning^12^. “SEM was performed under suitable accelerating voltage (20 kv) to minimized the charging effect. In addition, the tooth specimens contain inorganic components that provide sufficient conductivity, allowing clear imaging without metal coating”.

### Energy dispersive X-ray analysis (EDX) and mapping

Specimens of each group were used to evaluate the mineral content using an Environmental Energy Dispersive X-ray Analysis (EDX, Model Quanta 250, FEI business, Netherlands). The specimens were placed on aluminum stubs with the enamel surface facing upward within the closed chamber. The content of calcium, phosphorus and fluoride ions were then calculated in weight percent^22^.

### Confocal laser scanning microscopy (CLSM)

After inducing artificial enamel lesions via a demineralization protocol on previously prepared enamel specimens, the lower portion (approximately 1.5 mm) of a 3 × 3 mm window on one specimen from each group was coated with nail varnish to act as reference control. The lesions were then subjected to different treatment groups following the established protocol in our study. After the treatment phase, each specimen was immersed in a freshly prepared 0.1 mM Rhodamine B dye solution (C.I. 45170; Sigma-Aldrich^®^, Steinheim, Germany) for one hour. Excess dye was removed by thorough rinsing with phosphate-buffered saline (PBS) until no residual stain was detectable. The stained specimens were mounted on frosted glass slides using 80% glycerol, and coverslip edges were sealed to prevent dehydration. Image acquisition was performed using a confocal laser scanning microscope (Leica TCS SL, Leica Microsystems, Wetzlar, Germany) focused on the buccal surfaces of the specimens. Scanning was carried out at 10× magnification using an argon laser source set at 488 nm for excitation and an emission detection range of 498–514 nm. Fluorescence images were captured from both the untreated (control) and treated regions of each specimen and analyzed using the system’s dedicated software. Quantitative assessments of fluorescence depth were conducted to compare the lower (control) and upper (treated) halves of the lesion window in square microns (µm²), providing insight into the degree of remineralization^23,24^.

## Antibacterial activity assessment

The antimicrobial activity of the prepared formulas was evaluated against both commensal and cariogenic oral bacteria using Well diffusion method.

### Bacterial strains and cultivation conditions

The microbial species used in this investigation included *Streptococcus mutans* (ATCC 25175) and *Actinomyces viscosus* (ATCC 19246), both obtained from the Microbiological Resources Centre (MIRCEN), Cairo, Egypt. These strains were maintained at − 80 °C in cryogenic storage until use. Prior each experimental run, two successive subcultures were performed in tryptic soy broth (Difco, Sparks, MD, USA) to ensure active growth for both *Streptococcus* and *Actinomyces* species. All bacterial cultures were incubated aerobically at 37 °C for 24 h before use in assays^23^.

### Agar well diffusion method

The antibacterial efficacy of the tested formulations was evaluated using the agar well diffusion technique, as previously described by Karadağlıoğlu et al.^25^ with slight modifications. Briefly, freshly prepared bacterial suspensions of *Streptococcus mutans* (*S. mutans*) (ATCC 25175) and *Actinomyces viscosus* (*A. viscosus*) (ATCC 19246) were adjusted to match a 0.5 McFarland standard (approximately 1.5 × 10⁸ CFU/mL) and uniformly spread over Mueller–Hinton agar plates using sterile swabs. Wells (6 mm in diameter) were punched into the agar using a sterile cork borer, and 100 µL of each tested formulation was carefully pipetted into the wells. The plates were incubated at 37 °C for 24 h under aerobic conditions. After incubation, the diameter of the clear inhibition zones around each well was measured in millimetres using a digital calliper. Larger zones indicated greater antibacterial activity. The test was performed in triplicate to ensure reproducibility.

### Colony count method using spectrophotometry

A quantitative analysis of bacterial growth inhibition was conducted using a spectrophotometric colony count method. Bacterial cultures of *S. mutans* and *A. viscosus* were incubated with 1 mL of each test formulation in sterile tubes containing 9 mL of tryptic soy broth and incubated at 37 °C for 24 h. Following incubation, the optical density (OD) of the bacterial suspensions was measured at 620 nm using a UV–visible spectrophotometer (JENWAY 7315, UK). An optical density (OD) reading between 0.08 and 0.1 was considered equivalent to a 0.5 McFarland standard (approximately 1.5 × 10^− 7^ CFU/mL), or less than 300 colony-forming units (CFU)^22,26^. A reduction in OD values compared to the negative control group indicated antibacterial activity. Each measurement was carried out in triplicate^27^.

### Statistical analysis

Statistical analysis was performed using IBM^®^ SPSS^®^ Statistics for Windows (Version 20.0, IBM Corp., Armonk, NY, USA). Data were expressed as mean ± standard deviation (SD) for each experimental group. The normality of data distribution was assessed using the Kolmogorov–Smirnov and Shapiro–Wilk tests, which confirmed a parametric (normal) distribution of the data.

For comparisons involving repeated measurements within the same specimens, one-way repeated-measures analysis of variance (ANOVA) was applied to evaluate differences among more than two related groups. Pairwise comparisons between two related measurements were performed using the paired-samples t-test. For comparisons between independent experimental groups, one-way ANOVA was used, followed by Tukey’s post-hoc test for multiple comparisons. The level of statistical significance was set at *p* ≤ 0.05.

## Results

### Remineralization assessment

#### Surface Microhardness evaluation

As shown in Table 2, One-way repeated-measures ANOVA revealed a statistically significant effect of treatment stage on enamel surface microhardness for all groups (*P* < 0.001). A significant reduction in Vickers hardness values was observed in all groups after the demineralization phase compared with baseline values (*P* < 0.001). Following the remineralization protocol, all experimental groups exhibited a statistically significant increase in surface microhardness compared to their corresponding demineralized values (*P* < 0.001). However, the degree of hardness recovery varied according to toothpaste formulation. Intergroup comparison using one-way ANOVA demonstrated no statistically significant difference among groups at baseline (*P* > 0.05), indicating standardization of the enamel specimens before intervention. After remineralization, a statistically significant difference was detected among the tested groups (*P* < 0.05). Post-hoc Tukey analysis revealed that arginine-containing formulations (G4 and G5) showed significantly higher microhardness values compared to the non-arginine formulations (G2 and G3) (*P* < 0.05). Among all tested materials, the combined ginger and eggshell extract supplemented with 8% L-arginine (G5) demonstrated the highest mean surface microhardness values after remineralization, with statistically significant superiority over the commercial L-arginine toothpaste (G1) and the arginine free formulations (*p* < 0.05). No statistically significant difference was observed between groups G2; ginger extract and G3; ginger–eggshell formulations in the absence of L-arginine (*p* > 0.05), suggesting that the remineralization potential was markedly enhanced by L-arginine supplementation.

**Table 2:** Mean (± SD) Enamel Surface Microhardness (VHN) at Different Experimental Stages.

Group	Baseline	After demineralization	After remineralization
G1 Commercial L-arginine toothpaste	170.64 ± 36.40^A^ᵃ	78.28 ± 11.80^Bc^	163.87 ± 40.81^Ab^
G2 Ginger extract tooth gel	207.31 ± 49.93^A^ᵃ	83.01 ± 16.36^Bc^	187.07 ± 61.32^Abc^
G3 Ginger + eggshell extract tooth gel	198.27 ± 13.65^A^ᵃ	77.84 ± 6.47^Bc^	186.54 ± 62.19^Abc^
G4 Ginger + 8% L-arginine tooth gel	204.03 ± 36.56^A^ᵃ	79.55 ± 12.78^Bc^	207.76 ± 68.74^Aab^
G5 Ginger + eggshell + 8% L-arginine tooth gel	210.78 ± 14.57^A^ᵃ	72.17 ± 16.78^Bc^	222.96 ± 39.87^Aa^

Different lowercase letters within the same column indicate statistically significant differences between groups (one-way ANOVA followed by Tukey’s post-hoc test, *p* ≤ 0.05). Different uppercase letters within the same row indicate statistically significant differences between experimental stages within the same group (repeated-measures ANOVA followed by pairwise comparisons, *p* ≤ 0.05).

#### Scanning electron microscope (SEM) images analysis

**Fig. 1:**
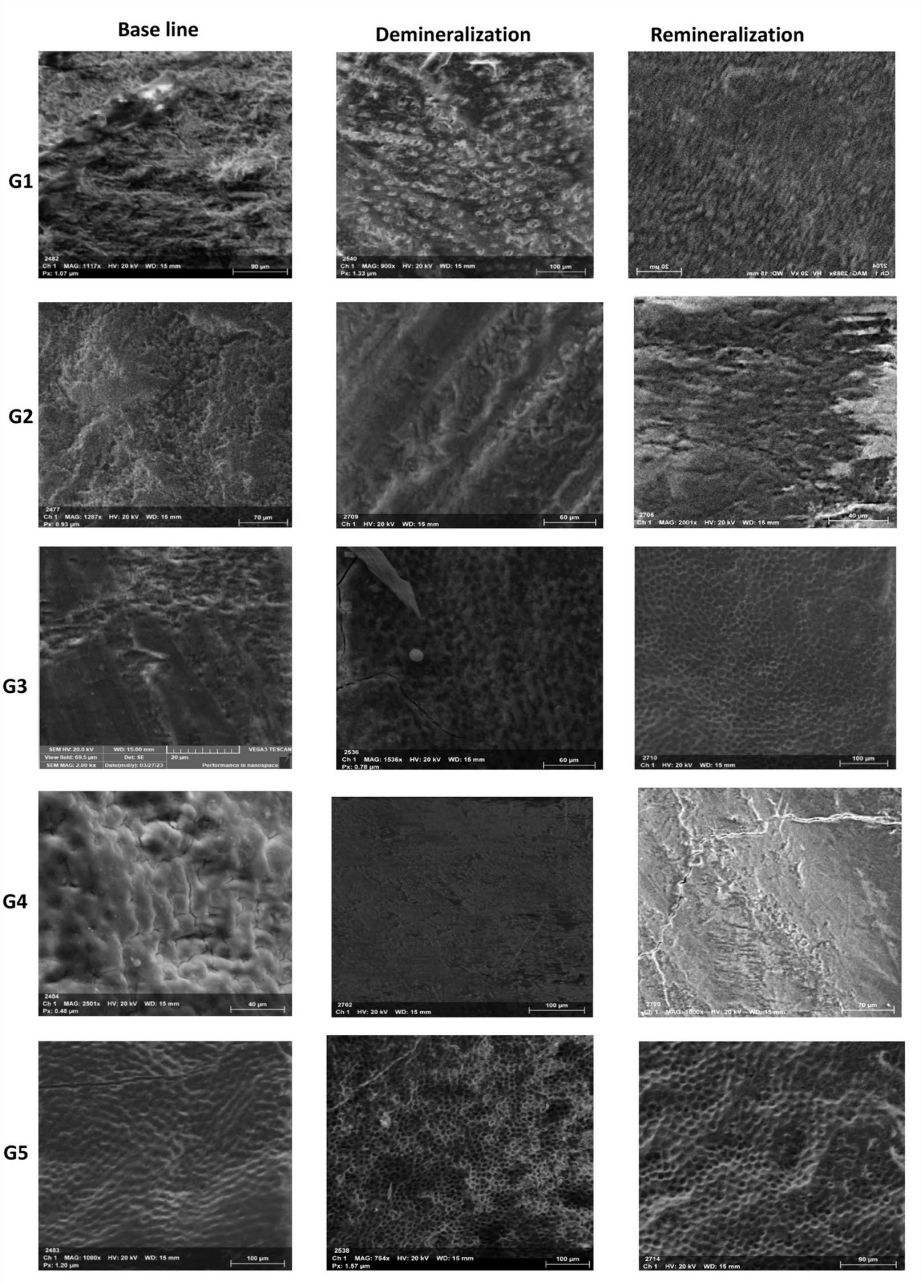
SEM of enamel specimens at baseline, after demineralization, and following the remineralization phase for Groups G1, G2, G3, G4, and G5. Baseline images show relatively compact enamel with variations in surface texture characteristic of bovine enamel. G1 and G5 showed the clearest prism-core exposure during demineralization, while G2 exhibited the greatest loss of interprismatic substance. G3 and G5 demonstrated the highest surface porosity in the demineralized state. After remineralization, G6 achieved the most uniform and compact mineral layer, followed by G3, whereas G1, G2, and G4 showed only partial or uneven surface recovery.

Figure 1, represented Scanning electron micrographs of enamel specimens at baseline, after demineralization, and following the remineralization phase for Groups G1, G2, G3, G4, and G5.

Baseline images show relatively compact enamel with variations in surface texture characteristic of bovine enamel.

After the demineralization stage, G1 and G5 exhibited the most pronounced etching patterns with clearly exposed prism cores forming a typical honeycomb structure. Increased surface porosity was particularly evident in G3 and G5, while G2 showed marked loss of interprismatic substance, resulting in widened prism boundaries and a rough etched appearance. G4 demonstrated generalized surface softening with reduced structural definition.

After the remineralization phase, partial mineral deposition with uneven surface recovery was observed in G1, G2, and G4, where prism outlines remained partially visible. In contrast, G3 and especially G5 showed more uniform mineral deposition, producing a smoother and more compact remineralized layer. Among all groups, G5 demonstrated the greatest restoration of enamel morphology, indicating superior remineralization effectiveness within the tested formulations.

#### Energy dispersive X-ray (EDX) analysis and mapping

Table 3 represented Energy Dispersive X-ray (EDX) analysis of enamel surface elemental composition (calcium (Ca), phosphorus (P), and fluoride (F)) in weight% (wt%) at baseline, after demineralization, and after remineralization for the control and different experimental groups.

**Table 3:** Energy Dispersive X-ray (EDX) analysis of enamel surface elemental composition; Ca, P and F by wt%.

Phase	Group	Ca (wt%)	*P* (wt%)	F (wt%)	*P* value
Baseline	G1	63.3 ± 2.9	31.4 ± 3.9	5.6 ± 2.7	0.112
	G2	73.3 ± 5.7	25.5 ± 6.0	0.4 ± 0.7	
	G3	68.1 ± 6.8	28.5 ± 9.9	3.4 ± 3.8	
	G4	66.2 ± 6.6	29.0 ± 10.0	5.0 ± 4.4	
	G5	66.2 ± 6.6	29.0 ± 10.0	5.0 ± 4.4	
Demineralization	G1	61.9 ± 6.9	36.6 ± 5.8	2.6 ± 1.1	0.031*
	G2	53.4 ± 5.4	38.1 ± 10.3	7.0 ± 3.5	
	G3	60.3 ± 7.5	34.2 ± 2.5	5.4 ± 7.0	
	G4	69.7 ± 1.2	30.3 ± 1.2	0.5 ± 0.7	
	G5	69.7 ± 1.2	30.3 ± 1.2	0.5 ± 0.7	
Remineralization	G1	59.2 ± 10.6ᵇ	36.9 ± 7.9ᵃ	6.4 ± 2.4ᵃ	< 0.001*
	G2	69.4 ± 2.9ᵃ	28.7 ± 2.6ᶜ	1.9 ± 2.5ᶜ	
	G3	66.2 ± 5.2ᵇ	29.6 ± 1.4ᵇᶜ	4.2 ± 4.6ᵇ	
	G4	69.4 ± 6.2ᵃ	29.6 ± 1.4ᵇᶜ	4.2 ± 4.6ᵇ	
	G5	69.4 ± 6.2ᵃ	29.6 ± 1.4ᵇᶜ	4.2 ± 4.6ᵇ	

Different superscript letters within the same column and within the same experimental phase indicate statistically significant differences among groups (one-way ANOVA followed by Tukey’s post-hoc test, *P* < 0.05). Absence of superscript letters indicates no significant intergroup differences. calcium (Ca), phosphorus (P), and fluoride (F).

The enamel surface elemental composition determined by Energy Dispersive X-ray (EDX) analysis is presented in Table 3. At baseline, no statistically significant differences were observed among the experimental groups for calcium, phosphorus, or fluoride content (*P* = 0.112), confirming adequate standardization and homogeneity of enamel specimens prior to treatment.

Following the demineralization phase, a statistically significant difference in elemental composition was detected among the groups (*P* = 0.031). This finding indicates variable degrees of mineral loss induced by the demineralizing challenge, as reflected by differences in Ca, P, and F weight percentages among the tested formulations.

After the remineralization phase, one-way ANOVA revealed a highly significant difference among groups for all measured elements (*P* < 0.001). Arginine-containing formulations (G4 and G5) exhibited significantly higher Ca weight percentages compared with arginine-free formulations, indicating enhanced mineral redeposition. Phosphorus levels showed moderate but significant intergroup variation, reflecting differences in mineral phase recovery. F content was highest in the commercial toothpaste group (G1), whereas the ginger-based arginine-free formulation (G2) demonstrated the lowest fluoride uptake. Overall, the EDX findings confirm that remineralization efficacy was strongly influenced by toothpaste composition, particularly the presence of arginine and calcium-rich components.

**Fig. 2:**
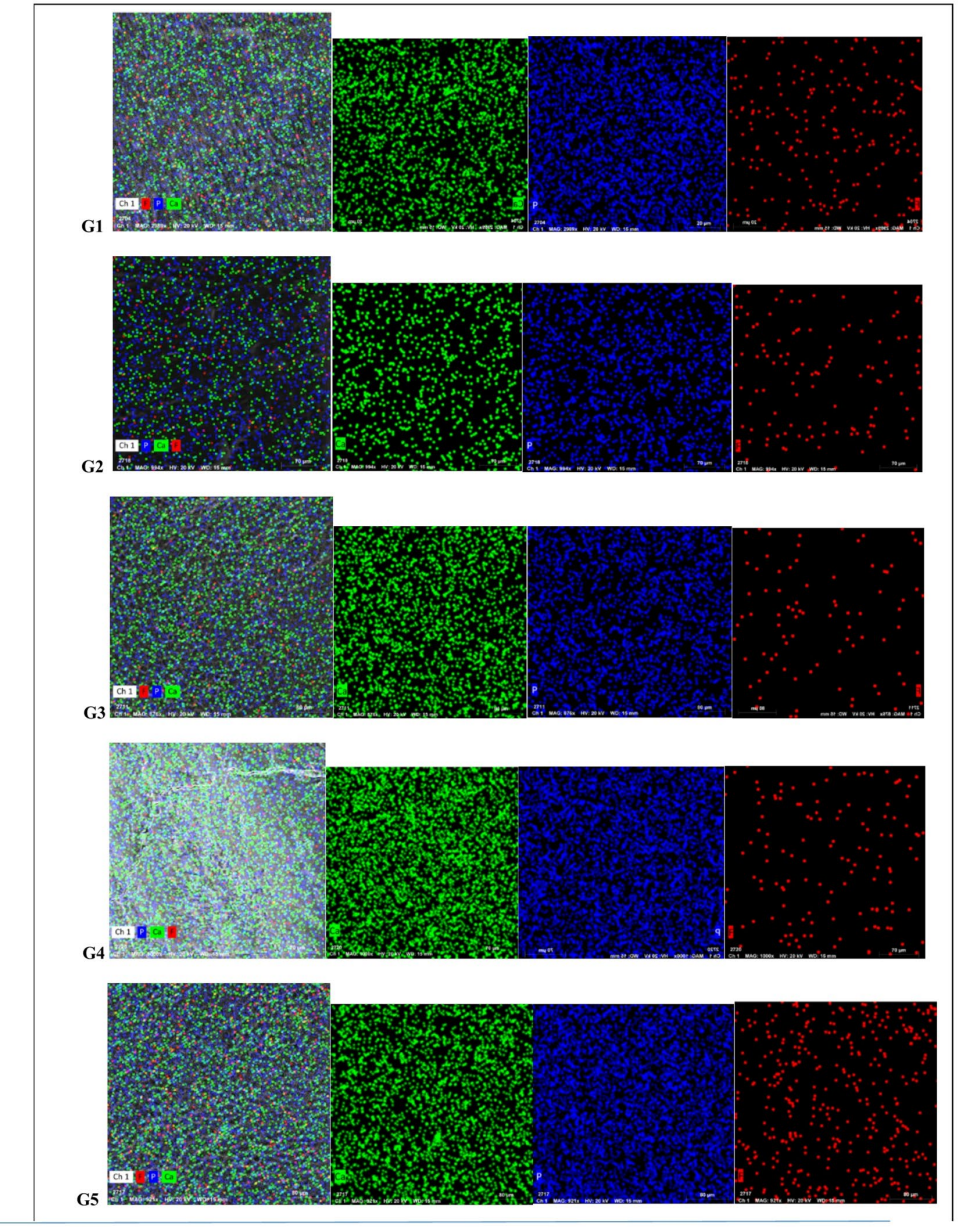
EDX mineral mapping of remineralized enamel surfaces showing the distribution of calcium (Ca, green), phosphorus (P, blue), and fluoride (F, red) across the different groups. G5 showed the strongest and most uniform Ca–P remineralization pattern, followed by G3and G4. G1 demonstrated moderate mineral deposition, while G2 produced weaker or less uniform patterns. Fluoride incorporation was low in all groups, with slightly higher presence in G1 and G5.

Figure 2 mineral mapping interpretation of remineralized enamel showing the distribution of calcium (Ca, green), phosphorus (P, blue), and fluoride (F, red) across the different groups. G1 demonstrates moderate Ca–P deposition with limited fluoride uptake. G2 shows good Ca and P distribution but minimal F. G3 exhibits strong Ca–P mineralization reflecting the effect of eggshell-derived hydroxyapatite. G4 shows uneven dense Ca–P deposition with low fluoride presence. G5 displays the most uniform and dense Ca and P distribution, indicating the highest remineralization potential among all groups, with slightly increased fluoride incorporation. Comparative mineral mapping after remineralization was summarized between the different groups in Table 4.

**Table 4:** Comparative mineral mapping summary after remineralization.

Group	Calcium (Ca) distribution	Phosphorus (*P*) distribution	Fluoride (F) distribution	Interpretation of remineralization quality
G1 Commercial Arginine	Moderate density, fairly uniform coverage	Uniform and consistent with Ca	Low, scattered points	Moderate remineralization with limited fluoride incorporation
G2 Ginger Extract	Moderate but somewhat less uniform	Strong, consistent P signal	Minimal; very sparse	Good Ca–P deposition but negligible fluoride uptake
G3 Eggshell + Ginger	High density; uneven distribution	Strong, uniform; follows Ca distribution	Very low	Strong Ca–P remineralization due to eggshell-derived hydroxyapatite
G4 Arginine + Ginger	High density; very uniform coverage	Present but irregular	Very limited	uneven dense remineralization; Ca–P present but less organized
G5 Arginine + Eggshell + Ginger	High density; most uniform surface coverage	Very strong and uniform, matches Ca signal	Low but slightly higher than natural-only groups	Best overall remineralization; superior Ca–P mineral integration

#### Confocal microscopy image analysis (CLSM)

Confocal laser scanning microscopy (CLSM) using Rhodamine B dye was employed to qualitatively assess enamel remineralization. Rhodamine B preferentially penetrates porous, demineralized enamel; therefore, greater red fluorescence intensity and depth indicate increased mineral loss, whereas reduced fluorescence reflects enhanced remineralization and pore occlusion.

**Fig. 3:**
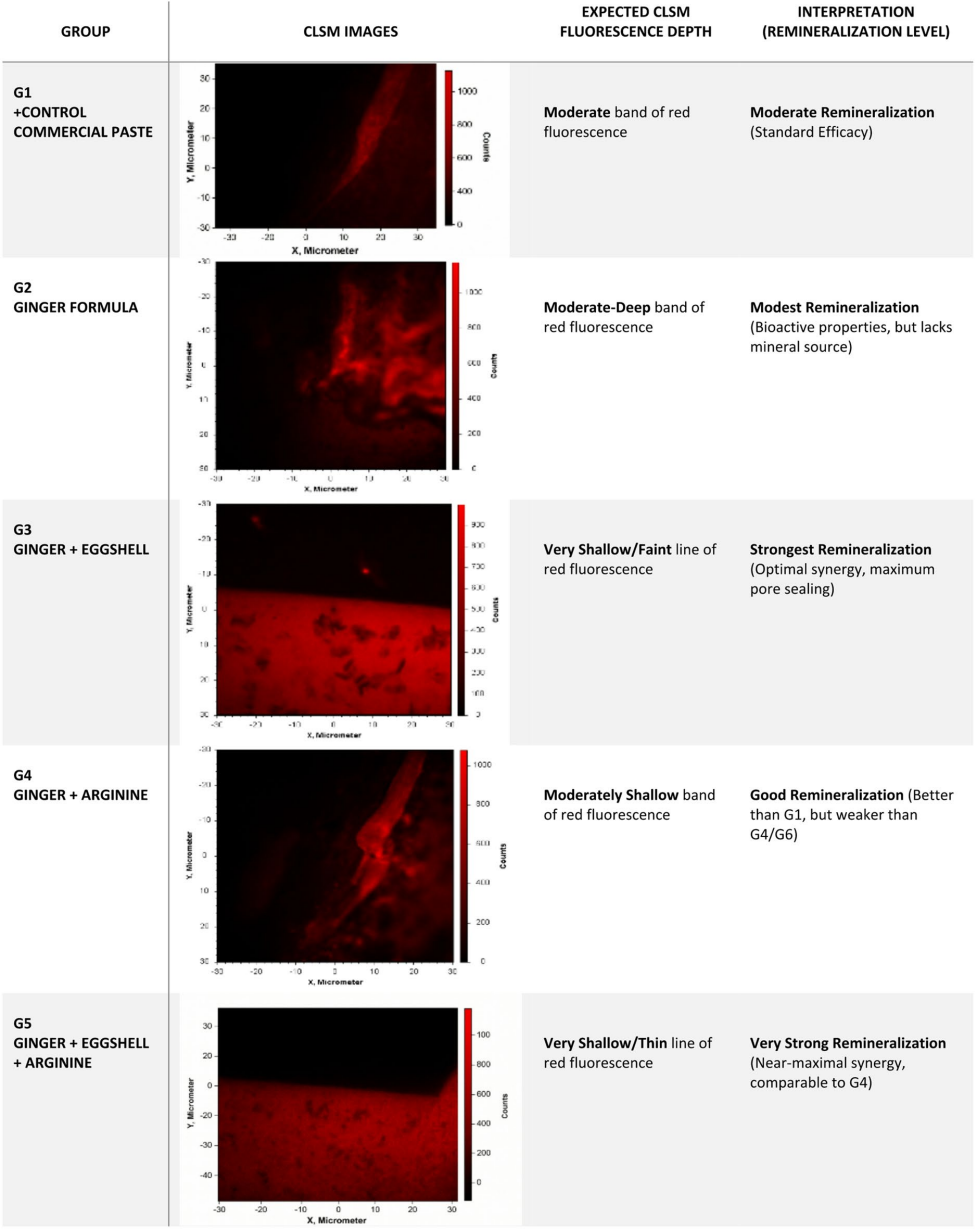
CLSM images for interpretation of remineralization level and fluorescence depth of the different groups.

CLSM images were represented in Fig. 3 which demonstrated distinct fluorescence patterns among the tested groups. The commercial toothpaste (G1) exhibited a moderate red fluorescent band, indicating a standard remineralization effect. The ginger extract formulation (G2) showed a moderate fluorescence intensity and depth, suggesting partial pore sealing with persistent enamel porosity. In contrast, the ginger–eggshell formulation (G3) displayed a marked reduction in fluorescence penetration with a more homogeneous fluorescence pattern, consistent with enhanced mineral deposition and effective pore occlusion. This finding reflects the synergistic remineralizing effect of eggshell-derived calcium combined with the bioactive properties of ginger. The ginger + arginine formulation (G4) demonstrated a reduced fluorescence depth compared to G2, indicating improved remineralization attributed to arginine’s alkalizing and mineral-promoting effect, although residual porosity remained due to the absence of a direct mineral source. The ginger–eggshell + arginine formulation (G5) exhibited the shallowest and thinnest fluorescent band, indicating minimal dye penetration and superior pore occlusion. This formulation showed the most consistent fluorescence reduction, reflecting the combined effect of calcium supply, pH buffering, and bioactive protection of the enamel surface.

### Antibacterial activity

The antibacterial efficacy of the commercial toothpaste and the experimental tooth gel formulations against *Streptococcus mutans* and *Actinomyces viscosus* is summarized in Table 5 One-way ANOVA revealed a statistically significant difference among the groups for both inhibition zone diameters and log bacterial counts (*P* < 0.05). In agar well diffusion assay, the arginine-containing formulations (G1, G4, and G5) produced significantly larger inhibition zones against both bacterial species compared with the arginine free formulations (G2 and G3) (Tukey’s post-hoc test, *P* < 0.05). The combined ginger–eggshell–arginine formulation (G5) exhibited antibacterial activity comparable to the commercial toothpaste (G1), with no statistically significant difference between the two groups (*P* > 0.05). In contrast the arginine free formulations; ginger-only (G2) and ginger–eggshell (G3) demonstrated moderate antibacterial effects characterized by smaller inhibition zones and higher residual bacterial counts. Quantitative spectrophotometric Colony Count (Log CFU) analysis confirmed these findings, as arginine-containing groups showed significantly lower log bacterial counts for both *S. mutans* and *A. viscosus* compared with arginine free formulations (*P* < 0.05). Overall, the results demonstrate a synergistic antibacterial effect when arginine was combined with ginger and eggshell extracts, with efficacy comparable to the commercial reference toothpaste.

**Table 5:** Combined antibacterial efficacy of tested toothpaste formulations against *Streptococcus mutans* and *Actinomyces viscosus*.

Groups and formulation	Inhibition zone (mm) S. mutans	Inhibition zone (mm) A. viscosus	Log bacterial count (S. mutans) (Log₁₀ CFU/mL)	Log bacterial count (A. viscosus) (Log₁₀ CFU/mL)
G1: Commercial paste (control)	21.0 ± 1.6^Aa^	32.2 ± 1.9^Aa^	7.1 ± 0.8^Bc^	5.7 ± 0.3^Bc^
G2: Ginger formula	12.6 ± 1.5^Cc^	20.4 ± 1.9^Cc^	12.6 ± 0.3^Aa^	9.2 ± 0.3^Ab^
G3: Ginger + eggshell formula	14.2 ± 1.3^Cc^	20.0 ± 1.6^Cc^	11.3 ± 0.9^Ab^	8.5 ± 0.7^Ab^
G4: Ginger + L-arginine formula	16.0 ± 2.6^Bb^	26.0 ± 1.4^Bb^	7.1 ± 0.8^Bc^	8.3 ± 0.7^Ab^
G5: Ginger + eggshell + L-arginine formula	19.6 ± 2.1^Aa^	29.4 ± 1.1^Aa^	7.9 ± 0.9^Bc^	7.1 ± 0.4^Bc^
values are expressed as mean ± standard deviation (SD). Different lowercase letters within the same column indicate statistically significant differences between formulations (one-way ANOVA followed by Tukey’s post-hoc test, *p* ≤ 0.05). Different uppercase letters within the same row indicate statistically significant differences between bacterial species within the same formulation (paired t-test, *p* ≤ 0.05). Lower Log bacterial counts (Log₁₀ CFU/mL) indicate greater antibacterial efficacy.				

### Correlation analysis between remineralization microhardness and antibacterial activity

In Table 6, Pearson’s correlation coefficient (r) was used to assess the relationship between mean enamel surface microhardness values after remineralization and corresponding antibacterial parameters across the five tested groups. Pearson’s correlation analysis revealed no statistically significant correlation between mean enamel surface microhardness values after remineralization and antibacterial outcomes for either *Streptococcus mutans* or *Actinomyces viscosus* (*P* > 0.05). Inhibition zone diameters against *S. mutans* (*r* = − 0.026, *P* = 0.968) and *A. viscosus* (*r* = − 0.011, *P* = 0.986) showed negligible correlations with remineralized enamel microhardness. Similarly, weak and non-significant correlations were observed between microhardness and log bacterial counts for *S. mutans* (*r* = − 0.183, *P* = 0.768) and *A. viscosus* (*r* = 0.280, *P* = 0.648). Pearson correlation analysis revealed weak positive correlations between antibacterial outcomes and mineral ion levels, suggesting only a modest relationship between remineralization indicators and bacterial reduction.

**Table 6:** Pearson’s correlation between enamel microhardness after remineralization and antibacterial parameters.

Antibacterial parameter	Pearson’s *r*	*P* value
Inhibition zone vs. *S. mutans* (mm)	− 0.026	0.968
Inhibition zone vs. *A. viscosus* (mm)	− 0.011	0.986
Log bacterial count (*S. mutans*)	− 0.183	0.768
Log bacterial count (*A. viscosus*)	+ 0.280	0.648

Negative r values indicate an inverse relationship, while positive values indicate a direct relationship. Statistical significance was set at *P* ≤ 0.05.

## Discussion

Growing concerns regarding dental fluorosis and the limited penetration of fluoride into deeply demineralized enamel have driven research toward safer, fluoride-independent remineralizing strategies^28^. In the present study, experimental natural bioactive tooth gel formulations were prepared and evaluated against a commercial L-arginine toothpaste for their remineralization and antibacterial efficacy on demineralized enamel. The selection of experimental groups in the present study was based on previous investigations, in which certain formulations did not demonstrate satisfactory stability or efficacy. Therefore, only the most promising combinations were included to allow focused and meaningful comparison of bioactive effects. To the best of our knowledge, this is the first study to comprehensively investigate combinations of ginger extract, eggshell-derived calcium, and L-arginine in a gel-based delivery system.

Gel formulations were selected to enhance contact time, reduce abrasiveness, and promote deeper mineral penetration compared with conventional toothpastes^29,30^. Remineralization was assessed using a multimodal approach combining surface microhardness testing, SEM, EDX with elemental mapping, and CLSM, providing complementary mechanical, morphological, chemical, and depth-related information^31–34^. Based upon our findings of this in vitro study, the null hypothesis was rejected as all of the experimental naturally based gel formulations could improve reminerlization of the demineralized enamel as well as inhibit the anti-cariogenic bacteria in comparison to the commercially used L- arginine tooth paste.

VMH testing showed a significant reduction in enamel surface microhardness after demineralization across all groups, confirming the validity of the artificial caries model. This decrease reflects acid-induced hydroxyapatite dissolution and mineral loss from the enamel surface and subsurface, consistent with previous in vitro study^35^. SEM findings corroborated the VMH results, revealing prism core exposure, increased surface porosity, and a characteristic honeycomb pattern—particularly in G1 and G5—indicative of preferential inter-prismatic enamel dissolution under acidic conditions^36,1^.

Following remineralization, all formulations produced a significant increase in enamel surface microhardness compared with their demineralized state, indicating mineral redeposition within the enamel matrix and supported the established relationship between surface microhardness and enamel mineral density^1,37^. However, hardness recovery was formulation-dependent, with arginine-containing groups (G4 and G5) showing significantly higher values than arginine-free formulations (G2 and G3), consistent with the role of L-arginine in pH elevation and promotion of calcium–phosphate precipitation^38,39^. The slightly higher pH observed in arginine-containing formulations may be attributed to its known alkalizing effect^38^. The ginger–eggshell formulation supplemented with 8% L-arginine (G5) achieved the highest microhardness recovery, exhibiting statistically superior performance compared with both the commercial L-arginine toothpaste and non-arginine experimental formulations. This enhanced effect might be attributed to the synergistic interaction between the calcium-rich eggshell-derived calcium carbonate, which served as a direct mineral source, and L-arginine, which facilitated mineral deposition by maintaining an alkaline microenvironment^40^. In contrast, no significant difference was observed between G2 and G3 in the absence of arginine, underscoring the necessity of pH-modulating and mineral-promoting agents for optimal remineralization^41^. SEM findings supported the VMH results, revealing partial and irregular surface recovery in G1, G2, and G4, more homogeneous mineral deposition in G3, and the most compact and smooth enamel surface in G5, consistent with enhanced and stable mineral precipitation reported for calcium-rich, pH-buffered systems^42,43^.

Elemental mineral mapping (Fig. 2) corroborated the quantitative EDX results by visualizing the spatial distribution of Ca, P, and F across remineralized enamel surfaces, confirming the reliability of the remineralization assessment and the influence of formulation composition on mineral content and deposition uniformity. The commercial L-arginine toothpaste (G1) showed moderate, homogeneous Ca–P deposition with limited fluoride incorporation, reflecting fluoride-mediated mineral stabilization rather than extensive mineral gain, consistent with previous reports describing fluoride’s role in enhancing enamel resistance rather than maximizing mineral gain^1^. The ginger-based formulation (G2) demonstrated continuous Ca–P coverage with minimal fluoride, suggesting mineral retention via organic matrix stabilization. However, the reduced fluoride signal indicated limited contribution to fluorapatite formation, which may explain the moderate remineralization efficacy observed in this group^44^. while the ginger–eggshell formulation (G3) exhibited dense and uniform Ca–P distribution, highlighting the effectiveness of eggshell-derived calcium as a direct mineral source which is strongly correlated with the increased calcium and phosphorus values obtained from EDX analysis. In the same context Saleh et al.^13^ proved that eggshell calcium facilitates hydroxyapatite re-precipitation, enhancing mineral density and surface integrity independently of fluoride availability. The arginine-supplemented ginger formulation (G4) showed dense but heterogeneous mineral deposition, likely due to pH-driven precipitation in the absence of sufficient calcium availability which promoted mineral precipitation but didn’t guarantee uniform crystal growth in the absence of an adequate calcium reservoir. Similar spatial variability had been reported in pH-modulating remineralization systems lacking sufficient calcium availability^38^.

In contrast, the combined ginger–eggshell–arginine formulation (G5) displayed the most uniform and intense Ca–P distribution with slight fluoride uptake, consistent with its superior EDX values and indicating a synergistic effect of calcium availability, pH modulation, and bioactive matrix protection. Collectively, these findings confirm that effective enamel remineralization depends on mineral availability, deposition uniformity, and physicochemical stability and can be achieved through fluoride-independent, calcium-rich, and pH-buffered formulations.

Recent investigations have confirmed that bioactive and biomimetic dentifrices significantly enhance enamel remineralization through ion release and surface mineral deposition mechanisms, resulting in improved microhardness recovery and structural repair. Furthermore, advanced dental materials promote remineralization via physicochemical interactions between calcium-phosphate ions and demineralized enamel surfaces^45^.

CLSM analysis confirmed enhanced remineralization when bioactive components were combined with effective mineral delivery systems. Similar to the synergistic mechanism reported by Epasinghe et al.^46^, where collagen stabilization and mineral delivery reduced lesion porosity and dye penetration, the present study demonstrated a comparable interaction between ginger bioactives, eggshell-derived calcium, and arginine-mediated pH modulation. CLSM images (Fig. 3) showed reduced dye penetration and thinner fluorescence bands in the ginger–eggshell and ginger–eggshell–arginine groups compared with ginger alone, indicating deeper remineralization. This suggests that while ginger contributes to matrix preservation and antibacterial effects, optimal remineralization requires its combination with a calcium source and pH-modulating agent to maximize mineral deposition.

Antibacterial assessment showed that all formulations significantly affected *Streptococcus mutans* and *Actinomyces viscosus*, with arginine-containing groups (G4 and G5) demonstrating significantly larger inhibition zones and lower log₁₀ CFU/mL values than arginine-free formulations (G2 and G3). Ginger-only and ginger–eggshell formulations exhibited moderate antibacterial activity, while spectrophotometric bacterial counts confirmed the superior efficacy of arginine-supplemented gels. This enhanced antibacterial effect is attributed to arginine-mediated pH elevation via arginolytic pathways, which suppress acidogenic bacteria and act synergistically with ginger phytochemicals and the buffering capacity of eggshell-derived minerals, in agreement with previous reports^38,47^, while ginger-derived compounds could contribute to additional antimicrobial activity^48^. No significant correlation was found between antibacterial outcomes and surface microhardness, indicating that remineralization and antibacterial effects are governed by distinct mechanisms. While remineralization depends mainly on mineral availability and pH modulation, antibacterial activity is driven by bioactive agents and alkalization^49^. This highlights the necessity of integrating independent remineralizing and antimicrobial strategies to achieve comprehensive caries prevention.

The correlation between antibacterial efficacy and mineral content was found to be weak, which was expected from a biological perspective. Enamel remineralization and antibacterial activity are governed by different mechanisms and influenced by multiple independent factors. While mineral deposition primarily depends on ion availability, surface interactions, and physicochemical conditions, antibacterial effects are mainly related to bioactive compounds, microbial susceptibility, and metabolic inhibition. Therefore, improvements in mineral composition do not necessarily occur in direct proportion to bacterial reduction. Similar observations have been reported in previous in vitro studies, highlighting the multifactorial nature of remineralization processes and biofilm modulation.

This *in vitro study* demonstrated that toothpaste formulation critically influences both enamel remineralization and antibacterial performance. Although all formulations partially restored enamel microhardness after demineralization, arginine-containing gels showed significantly superior remineralization, emphasizing the importance of pH modulation in mineral redeposition. The ginger–eggshell gel supplemented with 8% L-arginine achieved the highest efficacy, reflecting a synergistic interaction between eggshell-derived calcium, arginine-mediated alkalization, and ginger bioactives, as confirmed by mechanical, chemical, and microscopic analyses^50^. While ginger-based formulations alone provided moderate effects, optimal enamel repair and antibacterial activity required the combined presence of a mineral source and pH control, with remineralization and antibacterial actions operating through distinct yet complementary mechanisms.

It is worth mentioning that this in vitro study was employed, allowing strict control of environmental variables influencing enamel demineralization and remineralization. Multiple experimental formulations were comparatively evaluated against a commercial control, enabling direct assessment of the contribution of different bioactive components. Moreover, the study represents a comprehensive understanding of both remineralization potential and antimicrobial performance through combined mechanical, chemical, and microbiological analyses, including enamel surface microhardness evaluation, elemental analysis, and antibacterial testing. Additionally, the use of repeated treatment cycles simulating daily oral hygiene practices enhances the clinical relevance of the findings.

Nevertheless, some limitations should be considered since this study was conducted under controlled in vitro conditions that cannot fully replicate the complexity of the oral environment. Moreover, the absence of a dynamic pH-cycling model during the treatment phase might limit the direct clinical extrapolation despite the induction of initial demineralization using pH cycling. Additionally, the manually performed brushing protocol, despite standardization attempts, may introduce minor variability. Future studies incorporating dynamic cariogenic challenges are recommended. In addition, further in situ investigations and well-designed clinical trials are required to validate the efficacy, safety, and durability of these formulations under real oral conditions.

## Conclusions

This in vitro study supports the concept that fluoride-independent, bioactive tooth gel formulations incorporating calcium-rich sources and pH-buffering agents can effectively enhance enamel remineralization while simultaneously modulating cariogenic bacteria. Among the tested formulations, the combined ginger–eggshell–arginine tooth gel demonstrated the most favourable performance, suggesting its potential as a multifunctional caries-preventive approach.

## Data Availability

The datasets used and/or analyzed during the current study are available from the corresponding author upon reasonable request. All efforts were made to avoid compromising an individual’s privacy.

## Abbreviations

WSL: White spot lesion
VMH: Vickers microhardness
SEM: Scanning electron microscope
EDX: Energy dispersive X-ray analysis
CLSM: Confocal laser scanning microscopy
OD: Optical density
CFU: Colony-forming units
